# Multi-Year Phenotypic Assessment and Genetic Selection in Progeny Trials of *Liriodendron* Hybrids

**DOI:** 10.3390/plants15040638

**Published:** 2026-02-17

**Authors:** Yanghui Fang, Fuhui Liu, Tong Wang, Liang Fang, Jie Guo, Shunde Su, Xiaochou Chen, Libin Zhuang, Jie Sun, Daiquan Ye, Zhou Wang, Xuemei Wang

**Affiliations:** 1 College of Forestry and Grassland, Nanjing Forestry University, Nanjing 210037, China; 2 Fujian Provincial Forestry Science and Technology Promotion General Station, Fuzhou 350003, China; 3 Fujian Yangkou State-Owned Forest Farm, Nanping 353211, China; 4 Fuzhou Botanical Garden, Fuzhou 350012, China; 5 State Key Laboratory of Agricultural and Forestry Biosecurity, Fujian Agriculture and Forestry University, Fuzhou 350002, China; 6 College of Forestry, Fujian Agriculture and Forestry University, Fuzhou 350002, China; 7 Fujian Academy of Forestry Sciences, Fuzhou 350012, China

**Keywords:** *Liriodendron*, hybridization, family variation, long-term evaluation, early selection, breeding value, genetic gain

## Abstract

The conservation and genetic improvement of rare and endangered tree species are crucial for sustainable forest management. *Liriodendron chinense*, a relict species with limited distribution in China, exhibits high cross-compatibility with *Liriodendron tulipifera*, providing opportunities for interspecific hybrid breeding. In this study, 29 *Liriodendron* hybrids were established in a progeny trial plantation in Fujian Province, China, and subjected to multi-year evaluation of tree height, diameter at breast height (DBH), and individual stem volume. Significant differences (*p* < 0.01) among hybrids and hybrid × replicate interactions were detected for all traits across all assessment years, with individual stem volume showing the highest phenotypic coefficient of variation (35.30–40.56%). The mean annual increment in tree height increased during the early years, peaking at 1.50 m in the fourth year. Broad-sense and narrow-sense heritabilities for growth traits were consistently high (0.4073–0.7253 and 0.3410–0.6501, respectively), and the ratio of narrow-sense to broad-sense heritability ranged from 0.64 to 0.99, supporting the feasibility of early hybrid and individual selection. At a 10% selection intensity, hybrids No. 39, No. 59, and No. 74 were identified as elite, with selection based on individual stem volume providing the highest predictive accuracy and genetic gain (26.54–34.69%). Individual selection at a 1% intensity yielded genetic gains of 95.55–107.12% for stem volume. These results demonstrate substantial potential for early and efficient genetic improvement in *Liriodendron* hybrids, providing a theoretical foundation for the selection and deployment of elite hybrids and individuals in subtropical forest plantations.

## 1. Introduction

*Liriodendron chinense*, commonly known as the Chinese tulip tree, is a rare and ancient relict species in the *Magnoliaceae* family [1]. As one of only two extant species in the genus, alongside *L. tulipifera* from North America, *L. chinense* occupies a unique evolutionary position, with most of its congeners having vanished during the Quaternary glaciation [1]. It is widely used in urban landscaping due to its distinctive leaf shape and attractive flowers [2]. In addition, the species provides high-quality timber characterized by straight grain, fine texture, moderate density (air-dry density 0.45–0.55 g/cm^3^), and good workability, making it suitable for plywood, furniture, interior decoration, and papermaking [3]. The wood also has medicinal value [3].

Naturally, *L. chinense* is distributed in subtropical montane areas of China, often coexisting with broad-leaved deciduous or evergreen species at elevations of 700–900 m, within 22°–33° N and 103°–120° E [4]. In contrast, *L. tulipifera* is found in the lowland forests of eastern North America and exhibits high genetic diversity [1]. Both *L. chinense* and *L. tulipifera* are long-lived canopy trees, with lifespans commonly exceeding 100 years. Under favorable conditions, they can attain large dimensions at maturity. In China, *L. chinense* in old-growth stands has been reported to reach heights of >30 m, with typical mature DBH of 50–60 cm; exceptionally large individuals with DBH > 3 m have also been recorded in the core area of the Wuyishan National Nature Reserve. However, due to habitat destruction, biological constraints, and fragmentation caused by human activities, wild populations of *L. chinense* have declined significantly in recent decades [4]. The IUCN (International Union for Conservation of Nature) reports that *L. chinense* is now classified as Near Threatened [5,6], highlighting the need for targeted conservation and sustainable genetic management.

Significant interspecific genetic variation and high cross-compatibility between *L. chinense* and *L. tulipifera* provide a strong foundation for interspecific breeding. Since the 1960s, Chinese tree geneticists have advanced hybrid breeding in this genus, by overcome hybridization barriers, developing efficient propagation systems, and selecting superior hybrids, collectively known as “Sino-American tulip tree” (*L. sino-americanum*) [7,8]. Multi-site trials in Fujian, Jiangsu, Hubei, and Shandong have confirmed their growth advantages and adaptability. However, deployed varieties still suffer from a narrow genetic base and limited stress tolerance, emphasizing the necessity to broaden parent selection, construct multisource hybrid populations, and synchronously select elite hybrids and clones for enhanced growth and ecological adaptation [1,9,10]. Despite their successful cross-compatibility, *L. chinense* and *L. tulipifera* represent lineages that have been evolutionarily separated for millions of years. Published divergence-time estimates for the two species vary widely, ranging from approximately 4 million years to more than 28 million years, depending on datasets and calibration approaches [11,12,13]. The viability of these wide crosses is noteworthy and raises questions about the balance between potential heterosis/transgressive segregation and possible intrinsic incompatibilities.

Genetic diversity is fundamental to tree adaptability, evolutionary potential, and the stability and resilience of forest ecosystems [14]. In the context of accelerating biodiversity loss and climate change, elucidating heritability, sources of variation, and early selection efficiency is crucial for deploying elite individuals [5]. Long-term progeny testing and genetic parameter estimation provide the empirical basis for forest genetic improvement by quantifying heritability, sources of variation, and the efficiency of early selection across years [14,15]. Integrating molecular breeding with multi-site phenotypic assessment can further enhance genetic improvement efficiency and resilience in rare and endangered tree species [8].

The objective of this study was to quantify phenotypic and genetic variation, estimate key genetic parameters, and evaluate early selection efficiency and expected genetic gain in interspecific *Liriodendron* hybrids based on long-term progeny testing. Specifically, 29 interspecific or intraspecific hybrids of *Liriodendron* were established in a long-term progeny trial at Yangkou State Forest Farm, Fujian Province. Through multi-year assessments of tree height, diameter at breast height, and individual stem volume, we analyzed phenotypic and genetic variation, estimated genetic parameters, and performed early selection and genetic gain prediction for elite hybrids and individuals. These results provide a theoretical foundation for genetic improvement and new variety selection in *Liriodendron* hybrids, as well as for the conservation and sustainable utilization of rare forest resources.

## 2. Materials and Methods

### 2.1. Site Description

**Figure 1:**
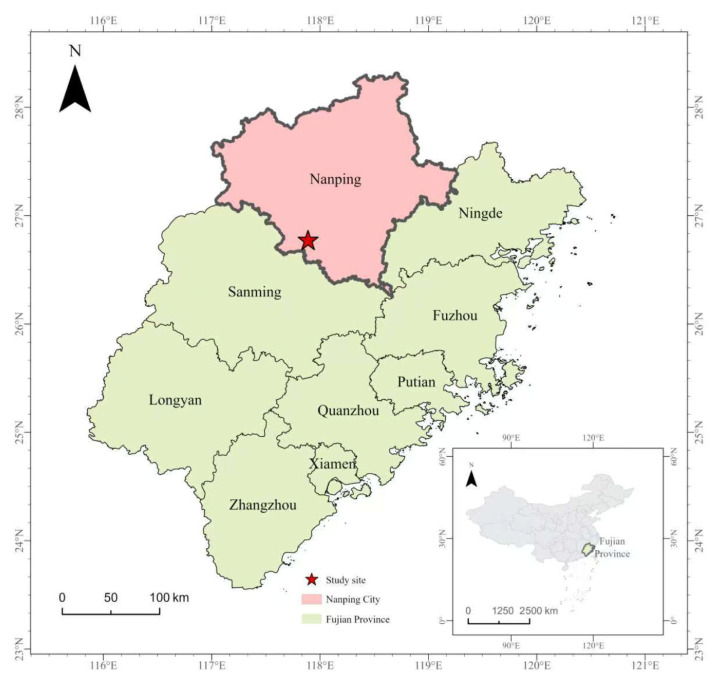
Geographic location of the study site (field trial) in Fujian Province, China.

The trial was established in the Nanshan Work Area of Yangkou State Forest Farm, Fujian Province, China (Figure 1). The geographic coordinates of the four corners are 117°53′24″ E, 26°46′27″ N; 117°53′22″ E, 26°46′26″ N; 117°53′20″ E, 26°46′28″ N; and 117°53′21″ E, 26°46′29″ N. The site is located in the low-hill extension zone of the Wuyi Mountains, in the northern mountainous region of Fujian, adjacent to the Futun River. Administratively, it falls under Jijie Village, Jianxi Town, Shunchang County, Nanping City, Fujian Province. The site has a typical southern subtropical monsoon climate, with an average annual relative humidity of 82%, annual precipitation of 1880 mm, an extreme temperature range from −6.8 °C to 40.3 °C, a mean annual temperature of 18.5 °C, and a frost-free period of 280 days.

### 2.2. Sapling Origin

The trial included 29 controlled-pollinated hybrid entries, comprising half-sib (male parent code ‘0’) and full-sib hybrids (Table 1). All 29 test hybrids originated from Nanjing Forestry University (Table 1). In May 2009, artificial controlled pollination of *Liriodendron* hybrids was conducted. Seeds were collected in October 2009 according to hybrid type, and hybrid-based sowing and sapling cultivation were carried out in spring 2010 at Qiaolin Forest Farm, Jiangsu Province.

### 2.3. Experimental Design

Afforestation was carried out in the spring of 2011 using a 4 m × 2 m spacing. A total of 29 hybrids were included, each arranged in 9-tree square plots, and three replicated blocks were set up (in total 27 saplings for each hybrid). A completely randomized block design was adopted to minimize environmental variation, with all plots arranged along the slope gradient.

### 2.4. Growth Trait Measurement

Tree height was measured in the winters of 2011, 2012, and 2013. Tree height (H) and diameter at breast height (DBH) were measured in autumn 2014, spring 2017, and spring 2025. Individual stem volume was calculated using *H* and DBH data. The formula for individual stem volume is [16]

*V* = 0.000050479055 × *D*^1.9085034^ × *H*^0.99076507^ (1)

where *D* is DBH in centimeters (cm); *H* is tree height in meters (m); *V* is individual stem volume in cubic meters (m^3^).

For reporting descriptive statistics, observations were summarized at the hybrid level: for each trait and measurement year, an across-block mean was calculated for each hybrid by averaging its plot means over the three replicate blocks.

### 2.5. Genetic Analysis

#### 2.5.1. Genetic Analysis Model

Excel was used for data organization. The linear model for analysis of variance is [15]

*Y*_ijk_ = *x* + *B_i_* + *F*_j_ + (B × F)*_ij_* + *E_ijk_* (2)

where *Y*_ijk_ is the observation for the *k*th tree of the *j*th hybrid in the *i*th replicate; $\bar{x}$ is the overall mean; *B_i_* is the effect of the *i*th replicate; *F_j_* is the effect of the *j*th hybrid; (*B* × *F*)*_ij_* is the interaction effect between the *i*th replicate and the *j*th hybrid; and *E_ijk_* is the residual. $\bar{x}$ and *B_i_* are fixed effects, and *F_j_*, (*B* × *F*)*_ij_*, and *E_ijk_* are random effects.

#### 2.5.2. Estimation of Genetic Parameters

Formulas for genetic parameter estimation are as follows [14]:

(3) $$\mathrm{Broad}\text{-}\mathrm{sense}\;\mathrm{heritability}:\;h_{f}^{2}=\;\delta_{f}^{2}/(\frac{\delta_{e}^{2}}{NB}+\frac{\delta_{\mathrm{fb}}^{2}}{B}+\;\delta_{f}^{2})$$

(4) $$\mathrm{Narrow}\text{-}\mathrm{sense}\;\mathrm{heritability}:\;h_{i}^{2}=4\delta_{f}^{2}/(\delta_{e}^{2}+\delta_{f}^{2}+\delta_{\mathrm{fb}}^{2})$$

(5) $$\mathrm{Genetic}\;\mathrm{value}:\;G_{i}=h_{f}^{2}(x-\bar{x})$$

(6) $$\mathrm{Breeding}\;\mathrm{value}:\;G_{a}=h_{i}^{2}(x-\bar{x})$$

(7) $$\mathrm{Genetic}\;\mathrm{gain}:\;∆G=\frac{\bar{G_{i}}}{\bar{x}}\times 100\%$$

where $\delta_{f}^{2}$ is the hybrid variance component; $\delta_{\mathrm{fb}}^{2}$ is the hybrid × replicate interaction variance component; $\delta_{e}^{2}$ is the environmental variance component; *B* is the number of replicates; *N* is the number of trees per plot; $\bar{G}$ is the mean genotypic value of selected individuals.

Variance components were estimated using the maximum likelihood method in IBM SPSS Statistics 19.0. The ratio of narrow-sense to broad-sense heritability, *I* =$\;h_{i}^{2}$/$h_{f}^{2}$, was used to measure the proportion of additive genetic variance to total genetic variance. A smaller *I* value indicates less additive genetic effect, which is unfavorable for selecting sexual breeding material but favorable for selecting clonal material; a larger *I* value indicates more additive genetic effect, which is advantageous for selecting sexual breeding material [14].

## 3. Results

### 3.1. Phenotypic Variation

Descriptive statistics for tree height, diameter at breast height (DBH), and individual stem volume of *Liriodendron* hybrid progenies across six measurements in the period from 2011 to 2025 are summarized in Table 2. Among the evaluated traits, individual stem volume consistently exhibited the highest phenotypic coefficient of variation (PCV), ranging between 35.30% and 40.56%, indicating substantial phenotypic diversity for this trait within the population.

**Figure 2:**
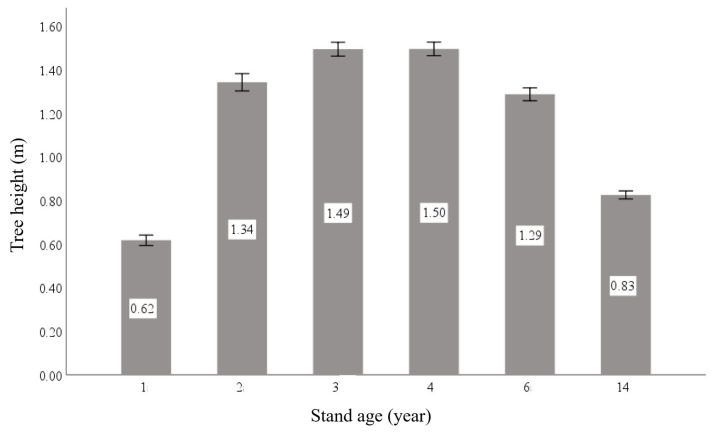
Average annual increment of tree height in the hybrid *Liriodendron* progeny test plantation. Error bars indicate the standard error of the mean.

Rapid early growth was observed in the progeny trials. Six years after planting, the mean tree height and DBH reached 7.72 m and 10.83 cm, respectively, providing a solid basis for early selection. Analysis of annual height increment (Figure 2) revealed a progressive increase in mean annual height growth during the first four years after establishment, peaking at 1.50 m in the fourth year. Thereafter, the annual increment declined gradually.

### 3.2. Genetic Variation

Analysis of variance revealed highly significant differences (*p* < 0.01) among hybrids and among block × hybrid interactions for tree height, DBH, and individual stem volume in all assessment years. This demonstrates the existence of true genetic variation and validates the effectiveness of early selection in this hybrid population, even in the year of planting.

All three traits exhibited relatively high genetic coefficients of variation in different years, with individual stem volume having the greatest values (26.46–30.68%) (Table 3), reflecting substantial genetic differentiation among hybrids. Broad-sense heritabilities for tree height, DBH, and stem volume were consistently high across years, indicating strong genetic control. Narrow-sense heritabilities were also high, and the ratio of narrow-sense to broad-sense heritability (*I* =${\;h}_{i}^{2}$/$h_{f}^{2}$) was generally greater than 0.64, with most values approaching 1 (most of the genetic variation was attributable to additive effects). This suggested that additive genetic effects predominate for these growth traits, making it feasible to achieve substantial genetic gains through hybrid and individual selection.

### 3.3. Hybrid Selection

Genetic selection was performed for tree height, DBH, and individual stem volume in all years at both 10% and 20% selection intensities. The selected hybrid IDs are presented in Table 4. There was high consistency among the optimal hybrids selected for different traits. As the improvement of stem volume is the primary breeding objective, hybrids selected based on 2024V (individual volume in 2024) were considered elite. At the 10% selection intensity, the best hybrids were No. 39 (MSL × EX), No. 74 (LS × MSL), and No. 59 (NK × SY), which were also top-ranked based on early height data.

At a 20% selection intensity, the consistency rates of selected hybrids based on early height, DBH, and stem volume with those identified by 2024V ranged between 50% and 83.3%. These results indicate that early selection of elite hybrids using data from the first six years after planting can achieve an accuracy rate of at least 50%, with stem volume providing the highest predictive accuracy. Early data on DBH and height were also effective for identifying superior hybrids for volume.

Mean genetic values and predicted genetic gains for selected hybrids at both selection intensities are shown in Table 5. The highest genetic gains were achieved for stem volume, with values ranging from 22.17% to 34.69%.

### 3.4. Individual Tree Selection

Individual selection was conducted for tree height, DBH, and stem volume at a 1% selection intensity among all surviving trees, with selected tree numbers, hybrid IDs, and genetic values detailed in Table 6. Using 2024V as the selection criterion, the most outstanding individuals were identified as No. 382 (hybrid 39), No. 027 (hybrid 6), No. 667 (hybrid 63), and No. 413 (hybrid 59). Notably, individual 027 showed superior stem volume as early as year six, while the others were classified as late-fast-growing types.

The genetic values, breeding values, and predicted genetic gains for selected individuals at 1% selection intensity are presented in Table 7. Selection for individual stem volume produced the highest genetic gains, ranging from 95.55% to 107.12%, illustrating the substantial potential for rapid genetic improvement through intense individual selection.

### 3.5. Differences in Growth and Survival Among Hybrid Types

In 2024, mean growth traits were broadly comparable among the three hybrid types (Table 8). Although interspecific hybrids showed slightly higher mean DBH and volume, the overall differences in growth among hybrid types were small. In contrast, survival differed markedly among hybrid types (Table 8). *L. tulipifera* intraspecific hybrids had a much lower survival rate in 2024 (25.46 ± 15.00%) than both *L. chinense* intraspecific hybrids (64.20 ± 17.48%) and interspecific hybrids (70.25 ± 11.72%).

## 4. Discussion

### 4.1. Genetic Parameters

The multi-year analysis of growth traits in *Liriodendron* hybrid progeny revealed significant differences among hybrids, with both the genetic variation coefficients and broad-sense heritability of tree height, DBH, and individual stem volume consistently high. This indicates that these traits are under strong genetic control. Our findings are consistent with previous studies, which also reported considerable genetic improvement potential for major growth traits [17,18,19]. Similarly, hybrid heritability for growth traits has been investigated [20], providing an important theoretical basis for hybrid selection and deployment.

Further analysis showed that the ratio of narrow-sense to broad-sense heritability (*I*) was high, indicating that additive genetic effects are predominant in the inheritance of growth traits in this population. Notably, *I* values close to 1 imply that genetic variance is largely additive (with relatively limited contributions from non-additive effects such as dominance or epistasis). The dominance of additive genetic variance is not only consistent with the general patterns observed in fast-growing broadleaf tree species [21], but also aligns with breeding progress seen in industrial conifers and eucalypts globally [14]. It has been emphasized that in populations where additive genetic variance predominates, traditional hybrid and individual selection methods can achieve substantial genetic gains [19,22,23]. In our study, the genetic variation coefficient for individual stem volume was particularly high across survey years, suggesting its suitability as a priority selection index.

It is worth emphasizing that this work benefits from a long-term, continuously surveyed progeny trial, allowing for a comprehensive assessment of hybrid differences and trait stability. Compared to studies that estimated genetic parameters from early or single-year data alone [22,24,25,26], our results provide a more robust and practically relevant basis for early selection and multi-generation genetic improvement.

### 4.2. Genetic Selection and Genetic Gain

Across different years and selection intensities, the optimal hybrids selected for tree height, DBH, and individual stem volume showed high consistency, providing further evidence for the feasibility of early selection. This effectiveness has also been reported in studies on other fast-growing tree species [14,19,27]. Strong genetic correlations among major growth traits allow early measurements to reliably predict later hybrid performance, as demonstrated in previous works [21,22,28,29]. Moreover, genetic correlations among measurement years and growth traits are crucial for evaluating early selection because they determine whether juvenile performance is predictive of later performance. In our previous study, correlations were consistently high across years and among growth traits (all >0.95), supporting the observed stability of hybrid ranking across selection ages and criteria [30]. Here, our results corroborate these findings; notably, when using individual stem volume as the selection criterion, the overlap and predictive accuracy of optimal hybrids surpassed those obtained from height or DBH selection.

In terms of genetic gain, selection of the top 10% and 20% hybrids by stem volume yielded predicted gains of 22.17% to 34.69%. Individual tree selection (top 1%) could achieve gains as high as 95.55% to 107.12%. These levels are comparable to those reported for other species in breeding programs [14,26,31,32]. Combined hybrid and within-hybrid selection has been shown to be most effective in populations dominated by additive genetic effects [21,22,33]. Thus, both hybrid and individual selection in *Liriodendron* hybrids are not only theoretically sound but also practically effective, offering significant prospects for genetic improvement.

Importantly, our ranking of superior hybrids and estimation of genetic gain are based on multi-year, multi-trait data, which reduces the influence of random environmental variation and enhances the stability and reliability of hybrid selection. This provides a solid genetic foundation for the breeding and deployment of improved *Liriodendron* hybrids. However, high predicted genetic gains at the juvenile stage and at a single site do not necessarily guarantee long-term trait stability across ages or across environments. Genetic gains may change when genotype-by-environment interactions are evaluated in multi-site trials or when rankings shift with age. In addition, predicted gains from individual-tree selection (e.g., the top 1%) may be upwardly biased when the effective sample size is small, because selection is based on a very small number of extreme individuals and is therefore more sensitive to sampling error and micro-environmental effects. Accordingly, the individual selection gains reported here should be interpreted cautiously and validated through continued measurements and, ideally, independent multi-site testing.

### 4.3. Hybrid Type Comparison

Although this study focuses on variation among hybrids, the question is whether any hybrid combinations show improvement over the parental species (*L. chinense* and *L. tulipifera*). This improvement could be expressed as heterosis, or even as performance exceeding the parental phenotypic ranges (transgressive segregation) [34,35]. Because the present trial did not include pure-species parental controls planted and measured under the same experimental design, we could not formally quantify mid-parent or better-parent heterosis. We also could not directly test whether hybrids exceeded parental ranges for height and diameter growth. Nonetheless, the 2024 results reveal a clear and biologically informative pattern: differences among hybrid-type groups are expressed much more strongly in survival than in juvenile growth. Mean height, DBH, and stem volume were broadly similar among the three hybrid-type groups, whereas survival of *L. tulipifera* intraspecific hybrids was substantially lower than that of *L. chinense* intraspecific and interspecific hybrids (Table 8). This suggests that, under the conditions of this site and at this juvenile stage, any “hybrid advantage” is more likely reflected in establishment success and stress tolerance than in markedly accelerated early growth.

Even without co-planted parental controls, the present dataset still allows a cautious within-trial interpretation of performance differences among cross types. Specifically, the fact that survival diverged strongly among hybrid types while mean juvenile growth differed little suggests that any putative “hybrid benefit” in this test is expressed primarily through establishment success and stress resilience rather than faster early growth. This pattern may reflect differences in establishment success and stress tolerance among genetic backgrounds and cross types, while early growth is constrained by site resources and stand development at the juvenile stage [36]. More direct evaluation of heterosis, transgressive segregation, and potential incompatibility effects will require co-planted parental controls and additional fitness-related indicators in future trials.

### 4.4. Limitations

Despite strict adherence to breeding trial protocols in hybridization design, hybrid selection, and progeny trial establishment, several limitations remain. For example, due to differences in parental compatibility and seed germination rates, the number of available hybrids and seedlings per hybrid did not fully meet the expectations of the initial design. Such issues are common in forest tree breeding programs, and improving hybridization efficiency and hybrid balance remains an important objective for future work [14,24,31].

Additionally, after the initial three years of plantation establishment, the reduction in management intensity led to a gradual decline in survival rates, with some hybrids showing significantly better survival than others. Full-sib hybrids generally exhibited greater ecological adaptability, which is consistent with findings in other tree species, highlighting the importance of optimizing parental combinations and screening for hybrid adaptability [19,26,28].

A further limitation is that this trial was conducted at a single site (Shunchang, Fujian province), which restricts our ability to assess genotype-by-environment interactions. Numerous studies have demonstrated that multi-site trials are essential for effective genetic improvement in forest trees, enabling the identification of broadly adapted superior hybrids [21,22,37,38,39]. We therefore recommend that future work incorporate multi-site progeny trials to improve the regional applicability and stability of selected *Liriodendron* hybrids. Such multi-site validation will also help determine whether the high predicted genetic gains observed here are maintained across environments and over longer-term growth.

Finally, this study primarily focused on juvenile field growth and survival and did not include reproductive fitness-related measures. Future work should quantify pollen viability, seed set (or seed yield), and seed viability, and integrate these with early seedling growth and survival to more comprehensively evaluate reproductive and early life performance. In addition, including co-planted parental controls under the same field conditions would enable more rigorous tests of mid-parent heterosis, better-parent heterosis, and transgressive segregation.

## 5. Conclusions

This study demonstrates that *Liriodendron* hybrids exhibit significant genetic variability for tree height, diameter at breast height, and stem volume, with additive genetic effects being predominant. Early selection based on multi-year growth data effectively identifies superior hybrids and individuals, particularly when stem volume is used as the primary selection criterion. The predicted genetic gains indicate a high potential for rapid improvement of key growth traits, providing a solid foundation for future selection and deployment of improved *Liriodendron* hybrids.

## Figures and Tables

**Table 1:** Hybrids tested in the trial and survival rates (%) during the period from 2011 to 2024.

No.	Hybrid ID	Female Parent	Male Parent	Survival Rate (%)					
				2011	2012	2013	2014	2017	2024
1	1	BK1	0	51.85	48.15	44.44	44.44	44.44	29.63
2	2	SY	0	74.07	70.37	74.07	66.67	62.96	44.44
3	6	BM3	0	66.67	66.67	66.67	66.67	70.37	22.22
4	7	BM4	0	55.56	55.56	55.56	51.85	44.44	11.11
5	9	LYS-2	0	51.85	55.56	51.85	51.85	51.85	33.33
6	10	LYS-3	0	22.22	22.22	22.22	18.52	18.52	7.41
7	11	LS3	0	81.48	88.89	88.89	88.89	85.19	74.07
8	12	LS4	0	55.56	55.56	51.85	51.85	51.85	48.15
9	14	WYS	0	85.19	88.89	88.89	77.78	77.78	55.56
10	15	BK	0	40.74	37.04	33.33	33.33	25.93	18.52
11	18	BK2	0	55.56	51.85	51.85	44.44	40.74	25.93
12	20	WYS	MSL	88.89	88.89	88.89	77.78	77.78	70.37
13	22	WYS	NK	100.00	100.00	100.00	100.00	96.30	85.19
14	31	MSL	WYS	83.33	83.33	83.33	83.33	83.33	61.11
15	39	MSL	EX	66.67	62.96	59.26	59.26	59.26	55.56
16	54	SZ	FY	85.19	85.19	81.48	81.48	85.19	77.78
17	59	NK	SY	85.19	85.19	85.19	85.19	70.37	77.78
18	63	LYS	WYS	92.59	92.59	92.59	88.89	81.48	66.67
19	65	LYS	LS	77.78	77.78	77.78	77.78	81.48	51.85
20	68	LYS	LS2	85.19	85.19	81.48	81.48	88.89	66.67
21	69	LYS	FY	81.48	81.48	81.48	81.48	77.78	51.85
22	71	LS	ZZY	85.19	88.89	88.89	88.89	81.48	55.56
23	72	LS	WYS	85.19	85.19	85.19	85.19	85.19	74.07
24	73	LS	SZ	85.19	85.19	85.19	85.19	81.48	77.78
25	74	LS	MSL	88.89	88.89	88.89	85.19	88.89	81.48
26	75	LS	LYS	96.30	92.59	92.59	92.59	92.59	81.48
27	77	LS	BK1	85.19	85.19	88.89	88.89	88.89	85.19
28	78	LS	FY	88.89	88.89	88.89	88.89	88.89	88.89
29	79	LS	F1	85.19	85.19	85.19	85.19	77.78	62.96

Note: Hybrids are identified by hybrid IDs, with maternal (female) and paternal (male) parent codes listed. Hybrids with male parent code “0” are open-pollinated half-sib hybrids (pollen parent unknown); hybrids with non-zero male parent codes are controlled-cross full-sib hybrids (pollen parent known). Parent code–species assignment: *L. tulipifera* includes BK, BK1, BK2, BM3, BM4, LYS, LYS-2, LYS-3, MSL, NK, SZ, ZZY, EX, and F1; *L. chinense* includes SY, LS, LS2, LS3, LS4, WYS, and FY. Cross-hybrid can be inferred from parental species: intraspecific when both parents are from the same species (or when the male parent is 0, based on the maternal species), and interspecific when the two parents are from different species. Survival rate (%) was calculated at the block (replicate) level first and then averaged across blocks. For each Hybrid ID and census year, survival in each block was computed as Survival (%) = (n_1_/n_2_) × 100, where n_1_ is the number of surviving seedlings in that block, and n_2_ is the number of planted seedlings in that block (n_2_ = 9). The survival rate reported here is the mean of the three block-level survival rates: Survival (%) = (Survival_1_ + Survival_2_ + Survival_3_)/3.

**Table 2:** Phenotypic variation analysis of growth traits among hybrids.

Trait	Hybrid-Level Minimum Value	Hybrid-Level Maximum Value	Hybrid-Level Mean	Hybrid-Level SD	Hybrid-Level Phenotypic CV (%)
2011H (m)	0.44	0.93	0.62	0.13	20.70
2012H (m)	1.98	3.75	2.68	0.43	15.99
2013H (m)	3.50	5.77	4.48	0.51	11.40
2014H (m)	4.84	7.42	5.98	0.67	11.24
2016H (m)	4.60	9.49	7.72	0.96	12.40
2024H (m)	8.40	14.12	11.55	1.38	11.96
2014D (cm)	5.81	9.93	7.47	1.17	15.70
2016D (cm)	8.14	13.33	10.83	1.53	14.17
2024D (cm)	10.68	20.17	16.09	2.46	15.28
2014V (m^3^)	0.0072	0.0313	0.0159	0.0065	40.56
2016V (m^3^)	0.0153	0.0707	0.0421	0.0148	35.30
2024V (m^3^)	0.0476	0.2372	0.1341	0.0496	37.02

Summary statistics are based on hybrid means. For each trait and measurement year, a mean was calculated for each hybrid across the three replicate blocks; Minimum value and Maximum value are the minimum and maximum of the 29 hybrid means, and Mean is the overall mean across the 29 hybrid means. Standard deviation (SD) and phenotypic coefficient of variation (CV) were calculated among hybrid means, where phenotypic CV = (SD/Mean) × 100. Units are given in parentheses in the column headings.

**Table 3:** Variance components and genetic parameter estimates for growth traits among hybrids.

Trait	Hybrid Mean	Variance Components			Genotypic CV (%)	$\mathrm{Broad-Sense}\;\mathrm{Heritability}\;h_{f}^{2}$	$\mathrm{Narrow-Sense}\;\mathrm{Heritability}\;h_{i}^{2}$	$h_{i}^{2}$ $⁄h_{f}^{2}$
		Hybrid	Rep × Hybrid	Error				
2011H	0.62 m	0.00690 **	0.00719 **	0.06686	13.40	0.5303	0.3410	0.6429
2012H	2.68 m	0.14073 **	0.04941 **	0.64103	14.00	0.7253	0.6773	0.9338
2013H	4.48 m	0.18095 **	0.13137 **	0.80100	9.50	0.6600	0.6501	0.9851
2014H	5.98 m	0.19919 **	0.41481 **	1.39331	7.46	0.4623	0.3969	0.8586
2016H	7.72 m	0.45158 **	0.77357 **	2.52213	8.70	0.5084	0.4820	0.9482
2024H	11.55 m	0.83148 **	2.00840 **	5.67620	7.89	0.4073	0.3905	0.9589
2014D	7.47 cm	0.78215 **	0.86843 **	3.79681	11.84	0.5926	0.5743	0.9691
2016D	10.83c m	1.28207 **	1.59858 **	7.50244	10.46	0.5425	0.4939	0.9105
2024D	16.09 cm	4.32995 **	1.81294 **	25.95151	12.93	0.5847	0.5397	0.9230
2014V	0.0159 m^3^	0.0000238 **	0.0000293 **	0.0001601	30.68	0.5405	0.4465	0.8261
2016V	0.0421 m^3^	0.0001241 **	0.0001567 **	0.0008506	26.46	0.5244	0.4387	0.8367
2024V	0.1341 m^3^	0.0016306 **	0.0010102 **	0.0133555	30.11	0.5034	0.4077	0.8100

In the trait codes, the number before the letter indicates the measurement year; H denotes tree height, D denotes diameter at breast height, and V denotes stem volume. For example, 2011H refers to tree height measured in 2011. Variance components: Hybrid, Rep × Hybrid, and Error represent the variance attributed to hybrid, replication × hybrid interaction, and residual error, respectively. Genotypic CV (%): coefficient of genotypic variation among hybrids; ** indicates significance at *p* < 0.01.

**Table 4:** Hybrid IDs selected under 10% and 20% selection rates based on genetic values for growth traits.

Trait	Selected Hybrid IDs (Top 10%)	Selected Hybrid IDs (Top 20%)
2011H	59, 39, 74	59, 39, 74, 22, 75, 72
2012H	59, 22, 20	59, 22, 20, 31, 74, 77
2013H	22, 59, 77	22, 59, 77, 39, 31, 75
2014H	59, 22, 77	59, 22, 77, 39, 74, 20
2016H	22, 77, 63	22, 77, 63, 11, 39, 74
2024H	59, 39, 22	59, 39, 22, 77, 14, 74
2014D	59, 22, 39	59, 22, 39, 77, 74, 20
2016D	77, 39, 74	77, 39, 74, 22, 63, 20
2024D	65, 39, 74	65, 39, 74, 59, 77, 22
2014V	59, 22, 39	59, 22, 39, 74, 77, 20
2016V	22, 63, 39	22, 63, 39, 74, 77, 65
2024V	39, 74, 59	39, 74, 59, 65, 14, 77

In the trait codes, the number before the letter indicates the measurement year; H denotes tree height, D denotes diameter at breast height (DBH), and V denotes stem volume. For each trait–year combination, hybrids were ranked by their genetic values, and selections were made under two selection intensities: 10% and 20%. Because the trial included 29 hybrids, the 10% and 20% selection rates correspond to selecting the top 3 and top 6 hybrids, respectively (rounded to the nearest whole number). Only the selected hybrid IDs are reported in the table, and the hybrid IDs are listed in descending order of genetic value (high to low).

**Table 5:** Genetic gain achieved by selection for growth traits.

Trait	Phenotypic Mean	Mean Genetic Value		Genetic Gain (%)	
		*sp* = 0.1	*sp* = 0.2	*sp* = 0.1	*sp* = 0.2
2011H (m)	0.62	0.0766	0.0639	12.36	10.30
2012H (m)	2.68	0.6020	0.4296	22.46	16.03
2013H (m)	4.48	0.6710	0.4662	14.98	10.41
2014H (m)	5.98	0.4797	0.3782	8.02	6.32
2016H (m)	7.72	0.7231	0.5756	9.37	7.46
2024H (m)	11.55	0.8946	0.7600	7.75	6.58
2014D (cm)	7.47	1.1219	0.9671	15.02	12.95
2016D (cm)	10.83	1.2210	1.0909	11.27	10.07
2024D (cm)	16.09	2.0657	1.7602	12.84	10.94
2014V (m^3^)	0.0159	0.00552	0.00438	34.69	27.54
2016V (m^3^)	0.0421	0.01117	0.00988	26.54	23.46
2024V (m^3^)	0.1341	0.03571	0.02973	26.63	22.17

In the trait codes, the number before the letter indicates the measurement year; H denotes tree height, D denotes diameter at breast height (DBH), and V denotes stem volume. For example, “2011H” refers to tree height measured in 2011. *sp* = selection proportion (*sp* = 0.1: top 10% of hybrids; *sp* = 0.2: top 20% of hybrids). Phenotypic mean is the mean value of the trait in the tested population. “Mean genetic value” is the average genetic value of selected hybrids. Genetic gain (%) is the percentage increase relative to the phenotypic mean.

**Table 6:** Individual No. (hybrid ID) selected under a 1% selection rate based on genetic values for growth traits.

Trait	Individual No. (Hybrid ID)
2011H	167 (63), 132 (39), 220 (74), 482 (75), 407 (59), 640 (39)
2012H	407 (59), 116 (22), 157 (59), 160 (59), 115 (22), 017 (2)
2013H	157 (59), 115 (22), 116 (22), 151 (59), 114 (22), 160 (59)
2014H	370 (22), 482 (75), 367 (20), 110 (22), 160 (59), 157 (59)
2016H	367 (20), 166 (63), 157 (59), 146 (59), 161 (59), 416 (63)
2024H	153 (59), 640 (39), 410 (59), 414 (59)
2014D	640 (39), 370 (22), 157 (59), 027 (6), 479 (75), 407 (59)
2016D	027 (6), 224 (74), 370 (22), 076 (14), 640 (39), 479 (75)
2024D	027 (6), 382 (39), 079 (14), 730 (74)
2014V	370 (22), 640 (39), 157 (59), 479 (75), 407 (59), 632 (31)
2016V	027 (6), 370 (22), 076 (14), 166 (63), 116 (22), 640 (39)
2024V	382 (39), 027 (6), 667 (63), 413 (59)

In the trait codes, the number before the letter indicates the measurement year; H denotes tree height, D denotes diameter at breast height (DBH), and V denotes stem volume. For each trait–year combination, surviving individuals were ranked by their genetic values, and selections were made under a 1% selection intensity. Only the selected entries are reported in the table as Individual No. (hybrid ID), and the entries are listed in descending order of genetic value (high to low). Because survival in 2024 was substantially reduced (Table 1), the 1% selection intensity in 2024 resulted in only four selected individuals.

**Table 7:** Selection response of individual trees for growth traits (1% selection intensity).

Trait	Phenotypic Mean	Mean Genetic Value	Mean Breeding Value	Genetic Gain (%)
2011H (m)	0.62	0.3967	0.6170	63.98
2012H (m)	2.68	1.4077	1.5075	52.52
2013H (m)	4.48	1.5646	1.5883	34.92
2014H (m)	5.98	1.063	1.2381	17.78
2016H (m)	7.72	1.8782	1.9808	24.33
2024H (m)	11.55	2.2063	2.3009	19.10
2014D (cm)	7.47	3.0323	3.1290	40.59
2016D (cm)	10.83	3.7059	4.0702	34.22
2024D (cm)	16.09	6.9136	7.4904	42.97
2014V (m^3^)	0.0159	0.01703	0.02061	107.12
2016V (m^3^)	0.0421	0.04023	0.04808	95.55
2024V (m^3^)	0.1341	0.13070	0.16136	97.49

In the trait codes, the number before the letter indicates the measurement year; H denotes tree height, D denotes diameter at breast height (DBH), and V denotes stem volume. For example, 2011H refers to tree height measured in 2011. Selection intensity = 1% (top 1% of individuals selected for each trait). Phenotypic mean: average trait value in the whole population. Mean genetic value: average estimated genetic value of selected individuals. Mean breeding value: average estimated breeding value of selected individuals. Genetic gain (%): percentage increase in trait value relative to the phenotypic mean.

**Table 8:** Growth traits and survival rates in 2024 by hybrid type.

	2024H (m)	2024D (cm)	2024V (m^3^)	Survival Rate in 2024 (%)
*L. chinense* hybrid	11.57 ± 1.50	14.76 ± 2.84	0.12 ± 0.06	64.20 ± 17.48
*L. tulipifera* hybrid	10.98 ± 1.62	15.91 ± 1.95	0.12± 0.06	25.46 ± 15.00
Interspecific hybrid	11.85 ± 1.19	16.72 ± 2.47	0.14 ± 0.05	70.25 ± 11.72

In the trait codes, the number before the letter indicates the measurement year; H denotes tree height, D denotes diameter at breast height, and V denotes individual stem volume. The 29 hybrids were assigned to three groups: *L. chinense* hybrid (intraspecific hybrids with *L. chinense* genetic background, including both open-pollinated half-sib hybrids with male parent coded “0” and controlled full-sib hybrids with known male parents, hybrid IDs: 2, 11, 12, 14, 72, 78, in total 6 hybrids); *L. tulipifera* hybrid (intraspecific hybrids with *L. tulipifera* genetic background, including open-pollinated half-sib hybrids with male parent coded “0”, hybrid IDs: 1, 6, 7, 9, 10, 15, 18, in total 7 hybrids); and interspecific hybrid (controlled full-sib hybrids derived from crosses between *L. chinense* and *L. tulipifera*, hybrid IDs: 20, 22, 31, 39, 54, 59,63, 65, 68, 69, 71, 73, 74, 75, 77, 79, in total 16 hybrids). See Table 1 for hybrid IDs and parent code–species assignment. Values are reported as mean ± SD calculated in two steps: (1) for each hybrid, trait values (H, D, V) were averaged at hybrid-level, and survival rate was calculated as the percentage of surviving trees at hybrid-level; (2) these hybrid-level values (29 hybrids) were then averaged within each of the three groups, with the accompanying standard deviation (SD) describing variation among hybrids within the group.

## Data Availability

The original contributions presented in this study are included in the article. Further inquiries can be directed to the corresponding authors.
